# Machine learning model for predicting the risk of AKI in early hemodynamically stable sepsis patients: a study based on the MIMIC IV database

**DOI:** 10.3389/fmed.2026.1846554

**Published:** 2026-05-26

**Authors:** Miao He, Xinran Li, Jiajing Wu, Luyao Huang, Nan Wang, Limin Chen, Mingxin Jiang, Zhe Chen, Lin Wei, Hong Zhang

**Affiliations:** 1 Department of Emergency Medicine, the First Affiliated Hospital of Anhui Medical University, Hefei, China; 2 Department of Emergency Medicine, Anhui Public Health Clinical Center, Hefei, China; 3 School of Life Sciences, Anhui Medical University, Hefei, China; 4 Department of Critical Care Medicine, Anhui Public Health Clinical Center, Hefei, China

**Keywords:** acute kidney injury, hemodynamic stability, machine learning, MIMIC-IV database, predictive models, risk factors, sepsis

## Abstract

**Background:**

The increasing prevalence and mortality of sepsis and acute kidney injury (AKI) pose significant threats to the survival of critically ill patients worldwide. Consequently, it’s essential to identify patients with sepsis-associated AKI early and to ascertain its risk factors. This study developed a machine learning (ML) model to identify patients at high risk of AKI among the hemodynamically stable sepsis.

**Methods:**

This study extracted clinical data from hemodynamically stable sepsis patients in the MIMIC IV Database for analysis, and collected clinical data from a hospital for external validation. The patients were randomly divided into a training set (70%) and a testing set (30%). Potential risk factors were screened out through univariate analysis and LASSO regression. Subsequently, models were constructed using Classification and Regression Tree, Gradient Boosting Machine, Logistic Regression, Random Forest, and Extreme Gradient Boosting (XGBoost). The performance of these models was assessed to determine the optimal predictive model.

**Results:**

A total of 8,276 hemodynamically stable sepsis patients were included in this study, among whom 3,061 patients (37%) experienced AKI. Nine risk factors were identified for model development using 70% of the data for training. By evaluating the model performance based on indicators including AUC, accuracy, sensitivity, kappa value, MCC, F1 score, Brier score, and DCA curve, the model constructed by XGBoost demonstrated the optimal performance. The external validation was consistent with the results.

**Conclusion:**

The XGBoost model exhibited optimal performance, and is suitable for predicting the risk of AKI in early hemodynamically stable sepsis patients.

## 1 Background

Sepsis is defined as a clinical syndrome characterized by life-threatening organ dysfunction caused by a dysregulated host response to infection (1). Sepsis frequently results in organ dysfunction. The overall incidence rate of sepsis accompanied by organ dysfunction among the intensive care unit (ICU) patients is 40.8 cases per 100,000 individuals. Moreover, the mortality rate of sepsis exceeds 40% (2–4). The development of multiple organ dysfunction syndrome (MODS) represents a critical complication of sepsis, substantially elevating the clinical mortality rate (5). Acute kidney injury (AKI) is a significant complication, increasing the risk of chronic issues (6). The concurrent occurrence of sepsis and AKI is associated with a substantially higher mortality rate (7).

Sepsis associated acute kidney injury (SA-AKI) is a severe complication of sepsis characterized by endothelial dysfunction leading to renal microvascular dysfunction. However, AKI also occurs regularly in clinically non-severe sepsis patients and is associated with heightened immune responses and increased mortality risk (8). Yet, fundamental differences exist in the pathogenesis between septic AKI and non-septic AKI (such as ischemic AKI). SA-AKI typically occurs independently of hypoperfusion, arising from mismatched metabolic demands and perfusion (9), and is mediated by concurrent pro-inflammatory and anti-inflammatory states. This condition activates in response to various pathogen-associated molecular patterns (e.g., endotoxin) and damage-associated molecular patterns (10). The primary distinction lies in hemodynamic changes. In contrast to other forms of AKI, direct measurements of overall renal blood flow during sepsis reveal an increase in flow under physiological conditions. In SA-AKI, the pathophysiology shifts from ischemia and vasoconstriction to congestion and vasodilation, and from acute tubular necrosis to acute tubular cell apoptosis or simple tubular cell dysfunction or desquamation (11, 12). Thus, even hemodynamically stable septic patients may still develop AKI. Current research predominantly focuses on septic shock patients. Identifying high-risk factors for AKI among patients with sepsis and stable hemodynamics in the early stages could facilitate the timely recognition of high-risk groups and enable the implementation of earlier and more targeted preventive and therapeutic interventions. This approach aims to reverse AKI at an early stage and further decrease AKI-related mortality. Currently, clinical diagnosis of AKI based on serum creatinine and urine output may exhibit significant delays and lack specificity.

As a significant branch of artificial intelligence, machine learning models exhibit enhanced adaptive capabilities and the capacity to address complex problems. Unlike traditional linear models, machine learning does not rely on linear relationships between input variables and outcomes, demonstrating superior performance in predictive accuracy (13, 14). In clinical practice, machine learning can efficiently process vast amounts of medical data, uncovering potential patterns and relationships. Previous studies have verified the effectiveness of transfer learning and multi-network feature fusion in medical image segmentation and classification (15). Recent studies indicate that machine learning models can effectively predict sepsis risk in retrospective cohorts, with high sensitivity (16). This study aims to develop and validate multiple ML models for predicting AKI risk in hemodynamically stable sepsis patients and to identify the model with optimal predictive performance.

## 2 Methods

### 2.1 Data sources used for analysis

The Medical Information Mart for Intensive Care(MIMIC)IV database is a publicly accessible multi-parameter ICU database provided by the Massachusetts Institute of Technology (MIT). It contains information on critically ill patients admitted to the ICU at Beth Israel Deaconess Medical Center in Boston, Massachusetts, United States, from 2008 to 2019. To apply for database access, we completed the online training course provided by the National Institutes of Health (NIH) and the Protection of Human Research Participants exam (ID: 66640220). In this study, we utilized PostgreSQL to extract demographic information, vital signs, comorbidities, laboratory test results, scoring data, and prognosis data from the MIMIC-IV database. All comorbidities were diagnosed based on International Classification of Diseases (ICD) codes from the 9th and 10th editions. Laboratory test results reflect the first recorded data during the ICU stay.

### 2.2 Inclusion and exclusion criteria

The occurrence of AKI is the primary outcome. Sepsis is defined based on the diagnostic criteria of the third international consensus definition of sepsis and septic shock (Sepsis-3), including suspected infection with a sequential organ failure assessment (SOFA) score ≥2 (17). The occurrence of AKI is defined as meeting the criteria established by the Kidney Disease Improving Global Outcomes (KDIGO) organization (18): (1) urine output < 0.5 mL/(kg·h) for ≥6 h, (2) serum creatinine increase ≥26.5 μmol/L within 48 h, (3) confirmed or presumed serum creatinine increase exceeding 1.5 times baseline within 7 days. Meeting any of these criteria establishes AKI diagnosis.

Inclusion criteria for this study: (1) sepsis diagnosis meeting Sepsis 3.0 criteria, (2) age ≥18 years. Exclusion criteria: (1) length of stay in the ICU < 24 h or non-first ICU admission, (2) missing the diagnosis of AKI, (3) missing data >20%, (4) complicated with chronic kidney disease (5), patients with hemodynamic instability:blood pressure is lower than 90/60 mmHg, or vasoactive drugs are needed to maintain blood pressure to a normal level.

### 2.3 Data statistics and analysis

Data analysis was performed using R software (version 4.5). For laboratory data with a missing rate <20%, multiple imputation methods (MICE) were employed for data completion, and we further conducted a comparative validation of model performance using two datasets: one with listwise deletion of missing values and the other with missing values handled by imputation. Median (interquartile range) was used to describe non-normally distributed continuous variables, analyzed with nonparametric tests. Normally distributed continuous variables were expressed as mean ± standard deviation (^−^x ± s), with intergroup comparisons performed using t-tests. Categorical variables were presented as percentages and analyzed using chi-square or Fisher’s exact tests. *p* value < 0.05 was regarded as statistically significant.

All patients included in the analysis were randomly assigned to a training set and a test set at a 70:30 ratio. To mitigate the effects of multicollinearity among variables, we employed LASSO technique to screen variables identified as statistically significant through univariate analysis. Key clinical baseline variables were retained on the basis of LASSO regression results, taking into account their clinical importance. These variables were integrated as the ultimate features into the machine learning models. Five machine learning algorithms—Classification and Regression Tree (CART), Gradient Boosting Machine (GBM), Logistic Regression (LR), Random Forest (RF), and Extreme Gradient Boosting (XGBoost) —were employed to construct predictive models. Ten-fold cross-validation resampling was employed to ensure the stability and reproducibility of model performance. The receiver operating characteristic (ROC) curves were plotted to assess model discrimination, with area under the receiver operating characteristic curve (AUC) serving as the primary metric for evaluating predictive performance. In addition to AUC, we considered accuracy (ACC), sensitivity, specificity, F1 score, kappa value and Matthews correlation coefficient (MCC) to comprehensively assess predictive performance, where values closer to 1 indicate better predictive capability. Furthermore, the Brier score was used to evaluate the overall performance of decision curve analysis, with a lower score indicating predictions closer to actual values. Decision curve analysis (DCA) was employed to evaluate the clinical utility of the model in decision-making. Ultimately, the selection of the optimal machine learning prediction model was primarily based on the AUC value, supplemented by various other statistical indicators. Model explainability analysis was further conducted using SHAP values to quantify the predictive contribution of each variable for acute kidney injury, which enhances the clinical interpretability and applicability of the predictive model.

At the same time, clinical data from the ICU of a hospital were collected to conduct external validation of the model and evaluate its clinical extrapolation value, which had been approved by the Clinical Medical Research Ethics Committee of our hospital (Registration Number: PJ-YX2022-015). We obtained informed consent forms from all the participants in the study. All research was conducted in accordance with the Declaration of Helsinki. The medical records and biological specimens utilized in this study were sourced from prior clinical diagnoses and treatments. Based on the relevant influencing factors identified in the initial phase, we retrospectively gathered clinical data from sepsis patients within 24 h of their admission to the ICU, applying the same inclusion and exclusion criteria.

## 3 Results

**Figure 1:**
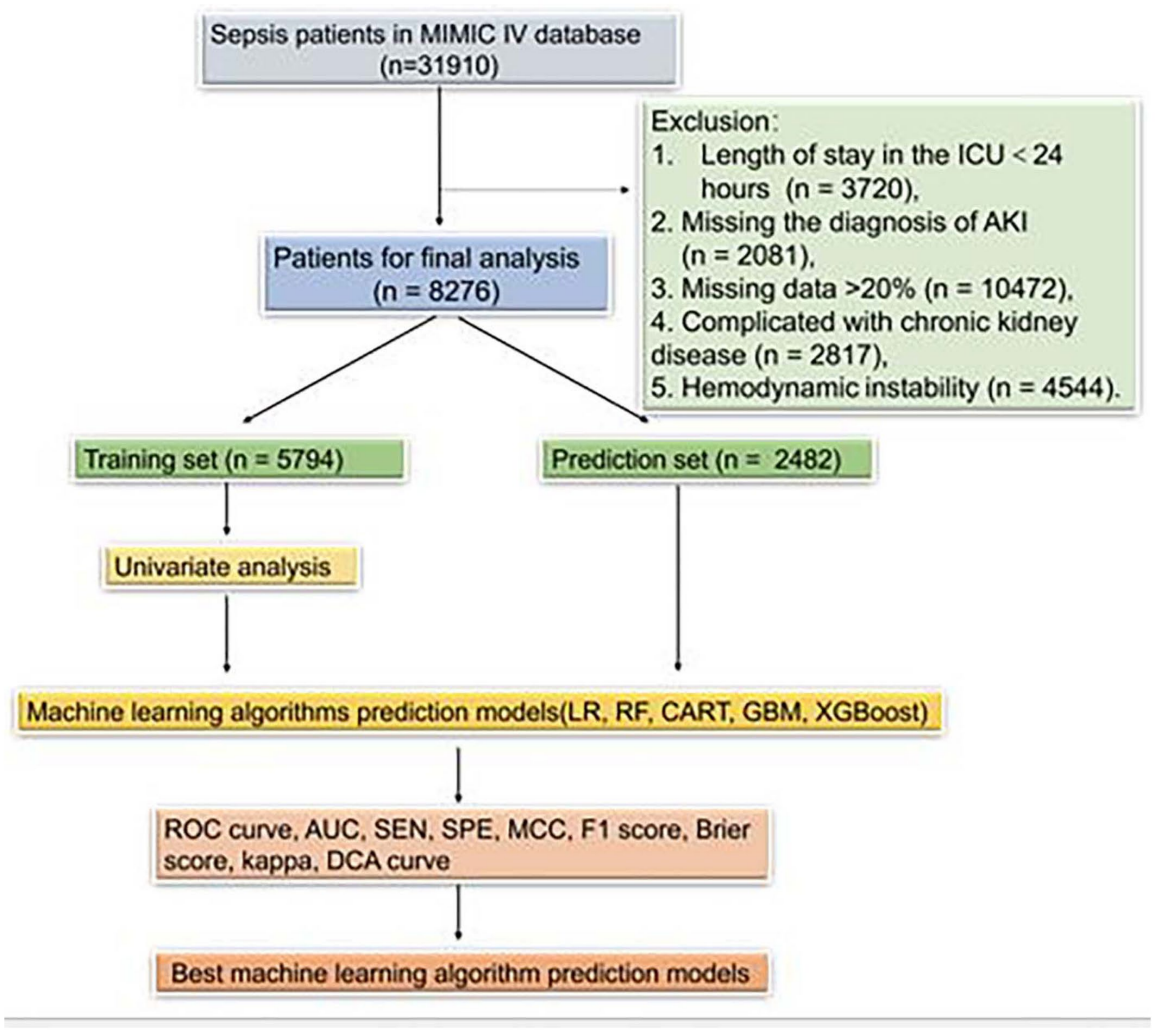
The flowchart of patient selection.

Based on inclusion and exclusion criteria, a total of 8,276 hemodynamically stable sepsis patients were ultimately enrolled in this study, among whom 3,061 patients (37%) developed AKI. The screening and analysis process is illustrated in Figure 1. All patients were randomly assigned to a training set (5,794 patients, 70%) and a testing set (2,482 patients, 30%). Thirty nine features were extracted from each patient based on clinical judgment and data availability, variable data with missing values less than 20% were filled in by multiple interpolation methods. Univariate analyses (including t-tests, nonparametric tests, and chi-square tests) were performed on the training set to determine whether relevant indicators differed statistically between AKI and non-AKI groups, p ≤ 0.05 is regarded as having a statistical difference (Table 1).

**Table 1:** Basic characteristics of the train data.

Subject_id	Non-AKI (*n* = 3,651)	AKI (*n* = 2,143)	*p* value
Age, year	66.00 (54.00, 76.00)	70.00 (58.00, 80.00)	<0.001
Gender(Male, %)	2,174 (59.5)	1,315 (61.4)	0.181
Weight, kg	79.40 (67.30, 94.60)	82.20 (68.60, 97.78)	<0.001
Underlying disease, yes (%)			
HTN	1729 (47.4)	816 (38.1)	<0.001
DM	910 (24.9)	773 (36.1)	<0.001
HLD	1,303 (35.7)	800 (37.3)	0.22
IHD	1,309 (35.9)	792 (37.0)	0.415
COPD	509 (13.9)	378 (17.6)	<0.001
Hematocrit, %	32.20 (27.70, 37.10)	31.00 (26.20, 36.20)	<0.001
Hemoglobin, g/dL	10.70 (9.20, 12.40)	10.10 (8.50, 12.00)	<0.001
Platelet count, K/uL	179.00 (128.00, 245.00)	178.00 (118.00, 248.00)	0.076
RDW, %	14.10 (13.20, 15.50)	14.90 (13.70, 16.70)	<0.001
Red blood cells, m/uL	3.53 (3.03, 4.13)	3.38 (2.86, 4.00)	<0.001
White blood cells, K/uL	11.50 (8.30, 15.40)	11.80 (8.30, 16.30)	0.045
Anion gap, mEq/L	13.00 (11.00, 16.00)	15.00 (13.00, 18.00)	<0.001
Glucose, mg/dL	127.00 (106.00, 158.50)	133.00 (109.00, 178.00)	<0.001
Potassium, mEq/L	4.10 (3.70, 4.50)	4.30 (3.80, 4.80)	<0.001
Lactate, mmol/L	1.70 (1.20, 2.50)	1.70 (1.20, 2.60)	0.036
pH	7.39 (7.34, 7.44)	7.37 (7.30, 7.42)	<0.001
PT, sec	14.10 (12.60, 16.10)	14.60 (12.80, 18.00)	<0.001
APTT, sec	30.00 (26.70, 35.30)	31.30 (27.50, 38.50)	<0.001
ALT, IU/L	26.00 (16.00, 53.00)	29.00 (16.00, 70.00)	<0.001
AST, IU/L	37.00 (24.00, 71.00)	45.00 (25.00, 109.00)	<0.001
Bilirubin_total, mg/dL	0.60 (0.40, 1.10)	0.70 (0.40, 1.40)	<0.001
Creatinine, mg/dL	0.80 (0.70, 1.00)	1.40 (1.10, 2.10)	<0.001
Urea nitrogen, mg/dL	16.00 (12.00, 22.00)	29.00 (20.00, 47.00)	<0.001
nbps, mmHg	120.00 (105.00, 138.00)	123.00 (107.00, 140.00)	0.003
nbpd, mmHg	66.00 (56.00, 79.00)	66.00 (56.00, 79.00)	0.539
SPO2, %	99.00 (96.00, 100.00)	98.00 (95.00, 100.00)	<0.001
Temperature, °C	98.30 (97.60, 99.00)	98.20 (97.60, 98.90)	0.041
Input amount, mL	4499.70 (2720.96, 6550.77)	3907.59 (2113.52, 6542.26)	<0.001
Ventilation, %	3,401 (93.2)	1934 (90.2)	<0.001
SOFA score	3.00 (2.00, 4.00)	3.00 (2.00, 5.00)	<0.001
SIRS score	3.00 (2.00, 3.00)	3.00 (2.00, 3.00)	0.021
Oasis	33.00 (28.00, 38.00)	34.00 (29.00, 40.00)	<0.001
GCS, score	15.00 (13.00, 15.00)	15.00 (13.00, 15.00)	0.144
APACHE II	17.00 (13.00, 21.00)	21.00 (17.00, 26.00)	<0.001
SA (%)	3,042 (83.3)	1,524 (71.1)	<0.001
GC (%)	973 (26.7)	695 (32.4)	<0.001

ICU, Intensive care unit; HTN, Hypertension; DM, Diabetes mellitus; HLD, Hyperlipidemia; IHD, Coronary heart disease; COPD, Chronic obstructive pulmonary disease; RDW, Red cell distribution width; PT, Prothrombin time; APTT, Activate partial thromboplastin time; ALT, Alanine aminotransferase; AST, Aspartate aminotransferase; SOFA, Sequential organ failure assessment; SIRS, Systemic inflammatory response syndrome; GCS, Glasgow coma scale; APACHE II, Acute physiology and chronic health evaluation II; SA, Sedative and analgesic drug; GC, Glucocorticoid.

**Figure 2:**
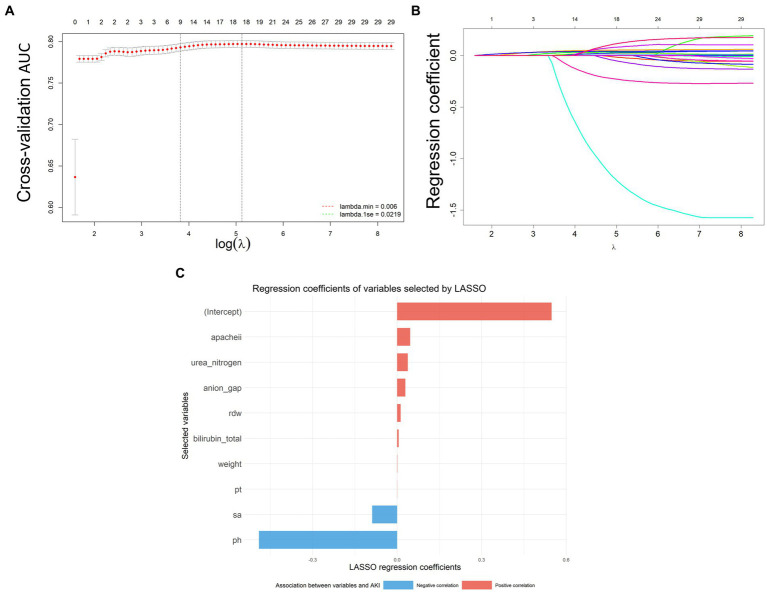
The potential risk factors were selected using the LASSO regression. **(A)** Graph of cross-validation results. The vertical line on the left side represents *λ* min, and the vertical line on the right side represents λ 1se. λ min refers to the λ value corresponding to the minimum mean squared error (MSE) among all λ values; λ 1se refers to the λ value corresponding to the simplest and best model obtained after cross-validation within a square difference range of λ min. **(B)** The coefficient path diagram of LASSO regularized binary logistic regression. The X-axis denotes the regularization parameter λ, while the Y-axis represents the logarithmic odds ratio coefficient of the independent variable. Each colored line corresponds to an independent variable, illustrating how the coefficient changes with λ. **(C)** Trend graph of variance filter coefficients. The red bar indicates a positive correlation, and the blue bar indicates a negative correlation.

To avoid multicollinearity among variables, we employed LASSO regression to filter variables included in the model. Results indicated that selecting a Lambda value of Lambda.1se (0.0219) filtered 9 features with non-zero coefficients (Figure 2): acute physiology and chronic health evaluation II (APACHE II) score, blood urea nitrogen (BUN), anion gap (AG), red cell distribution width (RDW), total bilirubin (TBIL), body weight, prothrombin time (PT), sedative and analgesic drug use (SA), and pH value. Based on the results of LASSO regression and considering clinical relevance, we further retained key clinical baseline variables including age, creatinine, and SOFA score. Finally, a total of 12 indicators were included for subsequent model training.

**Figure 3:**
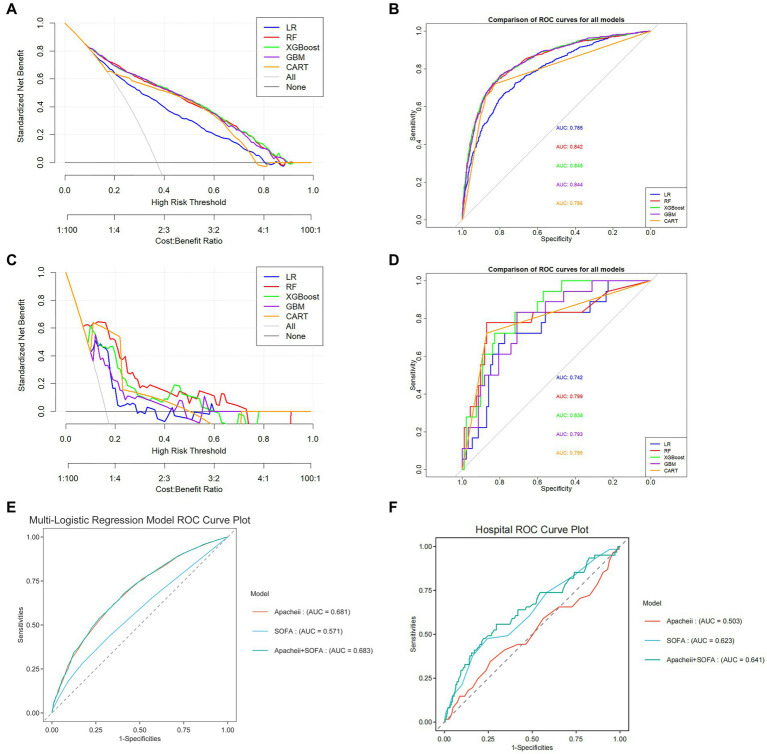
ROC curves and DCA curves of the ML models. **(A)** DCA curves of the internal validation ML models. The X-axis represents the threshold probability of clinical decisions, and the Y-axis represents the net benefit. The first horizontal axis represents the threshold probability value, and the second horizontal axis represents the loss-benefit ratio. A horizontal line indicates that all samples do not intervene, while a diagonal line indicates that all samples intervene. **(B)** ROC curve of the internal validation ML models. The X-axis represents the false positive rate (1 - specificity), and the Y-axis represents the true positive rate (sensitivity). AUC: Area under the curve. **(C)** DCA curves of the external validation ML models. **(D)** ROC curve of the external validation ML models. **(E)** ROC curves for traditional scoring systems in internal validation. **(F)** ROC curves for traditional scoring systems in external validation.

We constructed prediction models using five ML algorithms with the filtered indicators and compared model performance on both training and testing datasets. The performance comparison of the five models on the ROC curve is showed in Figure 3A. XGBoost demonstrated the highest predictive accuracy for the occurrence of AKI in patients with hemodynamically stable sepsis, achieving an AUC of 0.845. The subsequent models ranked as follows: GBM with an AUC of 0.844, and RF with an AUC of 0.842. LR recorded an AUC of 0.785, while CART achieved an AUC of 0.786. The X-axis of the DCA curve represents the threshold probability of clinical decisions, and the Y-axis represents the net benefit. The first horizontal axis represents the threshold probability value, and the second horizontal axis represents the loss-benefit ratio. A horizontal line indicates that all samples do not intervene, while a diagonal line indicates that all samples intervene. Within the threshold probability range of 0.1 to 0.7, all model curves are higher than the other two curves, suggesting that within this threshold range, the net benefit of using these models to guide clinical intervention is superior to “blindly intervening in all samples” or “not intervening in any samples”. These DCA curves are relatively close, indicating that the effects among the models are similar (Figure 3B). The detailed evaluation metrics for the five models were listed in Table 2. In summary, the XGBoost model demonstrated the best predictive performance, achieving the highest AUC (0.845), Kappa value (0.556), ACC (0.797), and MCC (0.558). The XGBoost-based model achieved higher net gains than the “full intervention” or “no intervention” strategies across the entire threshold range. Integration of the performance metrics indicates that the XGBoost-based model outperforms all other models.

**Table 2:** Performance comparison of the machine learning models.

Groups	Models	AUC	SEN	SPE	Kappa	ACC	F1	MCC	Brier
Internal validation	LR	0.785	0.878	0.512	0.414	0.743	0.811	0.426	0.178
	RF	0.842	0.852	0.684	0.544	0.790	0.837	0.544	0.151
	XGBoost	0.845	0.865	0.682	0.556	0.797	0.843	0.558	0.149
	GBM	0.844	0.856	0.694	0.556	0.796	0.841	0.557	0.150
	CART	0.786	0.877	0.651	0.544	0.794	0.843	0.548	0.161
External validation	LR	0.742	0.946	0.167	0.146	0.820	0.898	0.161	0.134
	RF	0.799	0.979	0.278	0.340	0.865	0.924	0.389	0.108
	XGBoost	0.838	0.936	0.278	0.253	0.829	0.902	0.263	0.121
	GBM	0.793	0.946	0.222	0.211	0.829	0.903	0.227	0.147
	CART	0.795	0.957	0.222	0.231	0.838	0.908	0.255	0.116

AUC, Area under the curve; SEN, Sensitivity; SPE, Specificity; ACC, accuracy; MCC, Matthews correlation coefficient.

Given that multiple imputation was applied for missing data handling, a sensitivity analysis was conducted using the unimputed dataset with listwise deletion of missing values to validate predictive performance. It was verified that the imputation procedure did not substantially alter the model results, indicating high robustness of the constructed model (Supplementary Table S1; Supplementary Figure S1).

To assess the reliability and clinical extrapolation capabilities of the model, we gathered data from sepsis patients in the ICU of the First Affiliated Hospital of Anhui Medical University: Anhui Provincial Public Health Clinical Center, followed by conducting external validation. A total of 370 sepsis patients were included in the external validation, Among them, 61 patients developed AKI. The specific performance indicators are presented in Table 2. Among the externally validated models, the AUC for XGBoost was the highest at 0.838, RF was 0.799, making it the second highest after XGBoost. Moreover, the other indicators such as SPE (0.278) and Kappa (0.253) were superior to other models. And the AUC and ROC curves for the external validation data are illustrated in Figures 3C,D. The external validation results were basically consistent with the model training results.

To further validate the predictive performance of the XGBoost model in comparison with traditional clinical scores, we additionally plotted the ROC curves of APACHE II and SOFA scores in both the training cohort and external validation cohort. In the test cohort, the AUC of APACHE II, SOFA, and the combination of APACHE II and SOFA was 0.681, 0.571 and 0.683, respectively. The corresponding AUC in the external validation cohort was 0.503,0.623, and 0.641, respectively, which were all lower than the AUC achieved by the XGBoost model (Figures 3E,F). The results demonstrated that the XGBoost model achieved superior predictive performance over conventional clinical scores, indicating favorable clinical significance.

**Figure 4:**
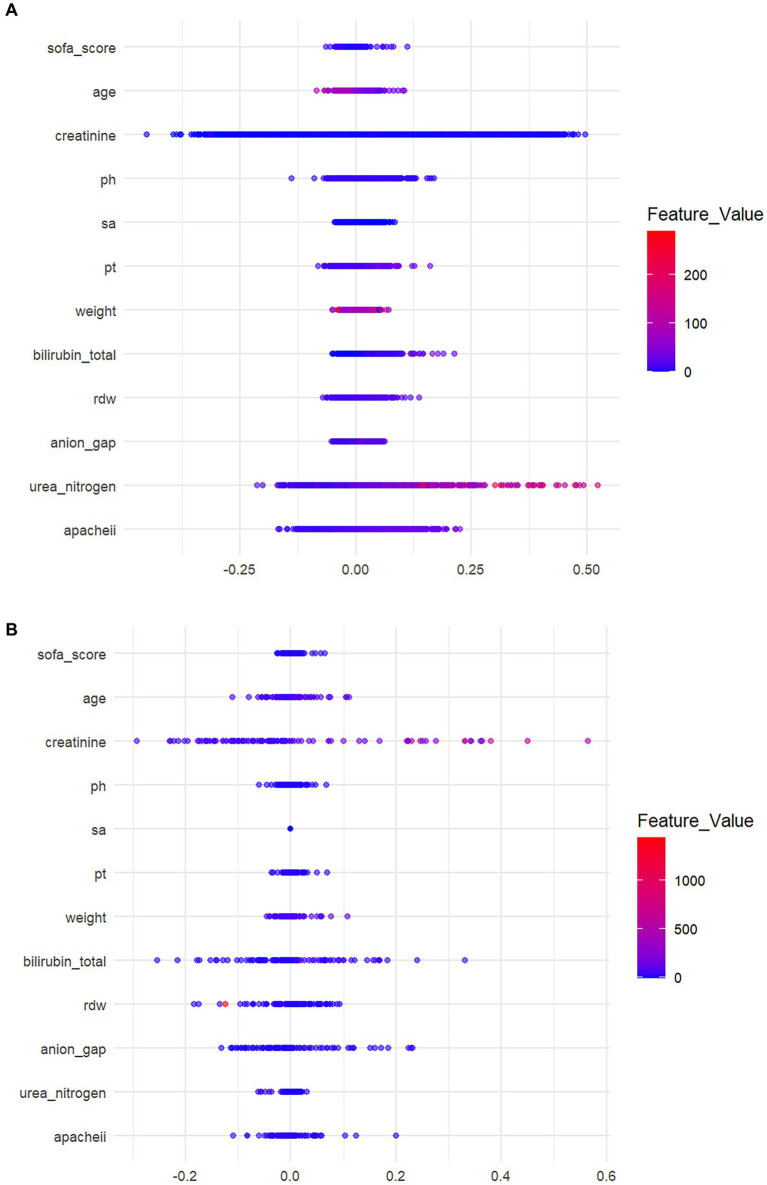
Summary plot of SHA*p* values for the model constructed by XGBoost algorithm: **(A)** Summary plot of SHAP values in the internal training set; **(B)** Summary plot of SHAP values in the external validation set. The vertical coordinates show the importance of the features. For the horizontal position ‘SHAP value’ shows whether the impact of the value is associated with a higher or lower prediction. The color of each SHAP value point indicates whether the observed value is higher (red) or lower (blue).

We further performed model explainability analysis using SHAP values to quantify the contribution of each variable to acute kidney injury prediction. In the internal validation cohort, SHAP analysis revealed that creatinine and urea nitrogen were the two core predictors, and elevated levels of both significantly increased the risk of adverse outcomes predicted by the model. APACHE II score, total bilirubin, and age also showed positive but weaker effects, while the impacts of other features were relatively limited (Figure 4A). In the external validation cohort, creatinine was the strongest predictor, followed by total bilirubin; increased levels of both markedly elevated the risk of adverse outcomes. Anion gap, age, and APACHE II score exerted moderate positive effects, and the influence of other variables was minimal (Figure 4B).

## 4 Discussion

This study successfully developed and validated a machine learning model using MIMIC IV data to predict the risk of AKI in hemodynamically stable septic patients. Machine learning has been widely applied to address medical and clinical challenges, emerging as a prominent research topic (19–21). Meta-ensemble learning, which combines multiple traditional machine learning and ensemble models, has been successfully applied to enhance early sepsis prediction performance (22). Here, we explored whether machine learning could enhance the prediction of AKI risk in hemodynamically stable septic patients and subsequently selected the model with the strongest predictive capability. This study analyzed data collected from the MIMIC IV database. Variables including APACHE II score, blood urea nitrogen, anion gap, RDW, total bilirubin, body weight, PT, the administration of sedative and analgesic drugs, pH, age, creatinine and SOFA score were employed as machine learning features for model construction. Five machine learning methods (GBM, LR, CART, RF, and XGBoost) were employed to construct models, which were subsequently evaluated for discrimination, fitness, and clinical efficacy. The findings indicate that the machine learning algorithms outperformed traditional linear regression, with XGBoost yielding particularly superior predictive results. The XGBoost model demonstrated balanced a relatively sensitivity and specificity, effectively optimizing the model’s predictive performance.

AKI represents a significant global public health challenge, characterized by elevated morbidity, mortality, and healthcare costs. It is a prevalent and severe complication among critically ill patients (23, 24). Apart from renal replacement therapy, limited interventions exist to enhance survival rates, mitigate renal impairment progression, or expedite renal function recovery. Prior research has predominantly concentrated on identifying risk factors for AKI onset in patients with septic shock. Nevertheless, in clinical settings, individuals with hemodynamically stable sepsis may also experience AKI, potentially resulting in delayed diagnosis and management. This study uniquely addresses the risk factors for AKI in hemodynamically stable sepsis patients, with the objective of facilitating early detection and prevention of AKI onset and progression.

Despite significant advancements in the epidemiology, pathophysiology, and diagnostic approaches for AKI in septic patients, the sensitivity and accuracy of AKI diagnostic criteria remain limited due to inadequate sensitivity and specificity of key indicators like urine volume and serum creatinine. Commonly utilized disease scoring systems like APACHE II, SOFA, and simplified Acute Physiology Score have demonstrated poor predictive performance for AKI (25). Although the emergence of data-derived clinical risk scores, renal imaging, functional assays and biomarkers has shown promise, they have not yet become part of a consensus definition or guideline (26). Nevertheless, they represent an opportunity to improve our diagnosis, assessment, treatment and prognosis of AKI. Currently, the appropriate indications and timing for medical interventions are still based on non-specific data from septic patients. The variables included in this study are prevalent across various clinical settings, facilitating the easy acquisition of relevant information.

The study findings reveal the association between classic indicators or scores such as urea nitrogen and APACHE II score with AKI occurrence. The APACHE II score is a standardized tool for assessing disease severity and predicting mortality risk in critically ill patients, widely used in prognostic evaluation and clinical decision support for ICU patients (27). When applied to AKI patients admitted to the ICU, they demonstrate good discrimination and calibration capabilities, serving as reliable prognostic indicators (28). Its advantage lies in its ease of use and more frequent application for risk stratification in AKI compared to other similar scoring systems. Additionally, Anion gap and pH are also significant influencing factors in this study. Anion gap acidosis results from the accumulation of organic anions caused by sepsis, diabetes, alcohol consumption, and various drugs and toxins, typically occurring upon admission to the ICU (29). A study indicated that elevated albumin-corrected anion gap (>20 mmol/L) is an independent risk factor for all-cause in-hospital mortality in SAKI patients (30). P Previous studies have demonstrated that metabolic acidosis is independently associated with an elevated risk of AKI in patients with chronic kidney disease (CKD) and may serve as a significant risk factor for AKI, CKD progression, and mortality (31). Additional studies have established RDW as a predictor of all-cause mortality in sepsis patients (32, 33). This study suggests that RDW also possesses a predictive capacity regarding the risk of AKI in sepsis patients. Furthermore, our findings indicate that total bilirubin levels constitute a risk factor for AKI in hemodynamically stable sepsis patients, whereas prior studies on whether total bilirubin levels represent a protective or risk factor for AKI remain controversial. A study using the MIMIC-III database examining the correlation between total bilirubin levels and neonatal AKI suggested it acts as a protective factor against AKI risk (34). However, other reports indicate that serum total bilirubin >2.0 mg/dL may promote AKI progression (35, 36). This study indicates that patients with higher body weight face an elevated risk of AKI development. Large-scale cohort studies involving the general population demonstrate increased mortality risk among overweight and obese individuals (37). Recent data from hospitalized patients and individuals with chronic diseases reveal a J-shaped relationship between body mass index (BMI) and mortality. Overweight and moderate obesity exhibit lower mortality rates compared to individuals with normal BMI or severe obesity (38, 39). We found an association between serum prothrombin time and the incidence of AKI. A prior investigation utilizing a machine learning prediction model for AKI related to cardiac surgery produced similar findings, indicating that preoperative blood urea nitrogen levels, prothrombin time, serum creatinine levels, total bilirubin levels, and age are positively correlated with AKI related to cardiac surgery (40). Furthermore, this study demonstrates the protective effect of sedative-analgesic drugs against AKI, aligning with the results of previous research. Increasing evidence in recent years indicates that dexmedetomidine can prevent AKI induced by sepsis, medications, surgery, and organ or tissue ischemia–reperfusion, highlighting its potential role in both the prevention and treatment of AKI (41–43).

Recent studies have increasingly employed machine learning techniques to predict AKI. Research has demonstrated that compared to traditional logistic regression models, the XGBoost models can better distinguish AKI subtypes with different volume responsiveness and identify significant clinical factors associated with AKI, such as age, urine creatinine concentration, maximum blood urea nitrogen concentration, and albumin (44). Another investigation revealed that machine learning models can accurately forecast maximum serum creatinine levels on the second and third days by utilizing a comprehensive array of demographic and physiological characteristics, thereby predicting AKI onset as defined by current clinical guidelines (45). In this study, machine learning algorithms achieved superior predictive performance compared to traditional logistic regression. Among all ML models, the XGBoost model demonstrated the best performance in AKI prediction, consistent with previous studies (46). A meta-analysis further revealed that the XGBoost model outperformed three other ML models (including LR, SVM, and RF) in predicting mortality among AKI patients (47).

The innovation of this study lies in the focus on the risk of AKI occurrence in hemodynamically stable sepsis patients—an area receiving limited research attention. We identified 12 relevant factors influencing the incidence of AKI and determined the most appropriate machine learning model, which outperformed conventional clinical scoring systems. This model is beneficial for clinical early warning and prevention of AKI based on these indicators. Furthermore, we not only employed data from the database but also gathered data from additional hospitals for verification to enhance the reliability. And the training set originates from Western countries, whereas the external validation set is sourced from China, demonstrating the applicability across diverse populations. Finally, the easy accessibility of the selected variables enhances the applicability across various medical fields and levels of the prediction model.

This study has several limitations. The training data were obtained from a single database, the external validation cohort was from only one hospital with a small sample size, and these factors may introduce selection bias and restrict generalizability to non-ICU sepsis populations. Furthermore, our model was developed using clinically accessible variables from the MIMIC-IV database. Given limitations in data availability, novel biomarkers and genetic polymorphisms were not included in the analysis. Although these indicators carry potential predictive value, they are not widely available in routine clinical settings. We prioritized readily measurable routine variables to strengthen real-world clinical applicability; future investigations will integrate additional novel biomarkers to further improve predictive performance.

## 5 Conclusion

The XGBoost model demonstrates superior performance in predicting the risk of AKI in patients with sepsis who exhibit stable hemodynamics during the early stages. This model may contribute significantly to guiding the early diagnosis and treatment of AKI.

## Data Availability

The original contributions presented in the study are included in the article/supplementary material, further inquiries can be directed to the corresponding author/s.

## References

[1] Evans L Rhodes A Alhazzani W Antonelli M Coopersmith CM French C . Surviving sepsis campaign: international guidelines for management of sepsis and septic shock 2021. Crit Care Med. (2021) 49:e1063–143. doi: 10.1097/CCM.0000000000005337, 34605781

[2] Hoste EAJ Bagshaw SM Bellomo R Cely CM Colman R Cruz DN . Epidemiology of acute kidney injury in critically ill patients: the multinational AKI-EPI study. Intensive Care Med. (2015) 41:1411–23. doi: 10.1007/s00134-015-3934-7, 26162677

[3] Peerapornratana S Manrique-Caballero CL Gomez H Kellum JA. Acute kidney injury from sepsis: current concepts, epidemiology, pathophysiology, prevention and treatment. Kidney Int. (2019) 96:1083–99. doi: 10.1016/j.kint.2019.05.026, 31443997 PMC6920048

[4] Markwart R Saito H Harder T Tomczyk S Cassini A Fleischmann-Struzek C . Epidemiology and burden of sepsis acquired in hospitals and intensive care units: a systematic review and meta-analysis. Intensive Care Med. (2020) 46:1536–51. doi: 10.1007/s00134-020-06106-2, 32591853 PMC7381455

[5] Tang F Zhao X Xu L Zhang J Ao H Peng C. Endothelial dysfunction: pathophysiology and therapeutic targets for sepsis-induced multiple organ dysfunction syndrome. Biomed Pharmacother. (2024) 178:117180. doi: 10.1016/j.biopha.2024.117180, 39068853

[6] Zarbock A Koyner JL Gomez H Pickkers P Forni L. Sepsis-associated acute kidney injury-treatment standard. Nephrol Dial Transplant. (2023) 39:26–35. doi: 10.1093/ndt/gfad142, 37401137

[7] Bouchard J Acharya A Cerda J Maccariello ER Madarasu RC Tolwani AJ . A prospective international multicenter study of AKI in the intensive care unit. Clin J Am Soc Nephrol. (2015) 10:1324–31. doi: 10.2215/CJN.04360514, 26195505 PMC4527019

[8] Murugan R Karajala-Subramanyam V Lee M Yende S Kong L Carter M . Acute kidney injury in non-severe pneumonia is associated with an increased immune response and lower survival. Kidney Int. (2010) 77:527–35. doi: 10.1038/ki.2009.502, 20032961 PMC2871010

[9] Schrier RW Wang W Poole B Mitra A. Acute renal failure: definitions, diagnosis, pathogenesis, and therapy. J Clin Invest. (2004) 114:5–14. doi: 10.1172/JCI22353, 15232604 PMC437979

[10] Morrell ED Kellum JA Pastor-Soler NM Hallows KR. Septic acute kidney injury: molecular mechanisms and the importance of stratification and targeting therapy. Crit Care. (2014) 18:501. doi: 10.1186/s13054-014-0501-5, 25575158 PMC4729166

[11] Rector F Goyal S Rosenberg IK Lucas CE. Sepsis: a mechanism for vasodilatation in the kidney. Ann Surg. (1973) 178:222–6. doi: 10.1097/00000658-197308000-00021, 4723431 PMC1355637

[12] Wan L Bagshaw SM Langenberg C Saotome T May C Bellomo R. Pathophysiology of septic acute kidney injury: what do we really know? Crit Care Med. (2008) 36:S198–203. doi: 10.1097/CCM.0b013e318168ccd5, 18382194

[13] Beam AL Kohane IS. Big data and machine learning in health care. JAMA. (2018) 319:1317–8. doi: 10.1001/jama.2017.18391, 29532063

[14] Lai W Kuang M Wang X Ghafariasl P Sabzalian MH Lee S. Skin cancer diagnosis (SCD) using artificial neural network (ANN) and improved gray wolf optimization (IGWO). Sci Rep. (2023) 13:19377. doi: 10.1038/s41598-023-45039-w, 37938553 PMC10632393

[15] Ghafariasl P Zeinalnezhad M Chang S. Fine-tuning pre-trained networks with emphasis on image segmentation: a multi-network approach for enhanced breast cancer detection. Eng Appl Artif Intell. (2025) 139:109666. doi: 10.1016/j.engappai.2024.109666

[16] Fleuren LM Klausch TLT Zwager CL Schoonmade LJ Guo T Roggeveen LF . Machine learning for the prediction of sepsis: a systematic review and meta-analysis of diagnostic test accuracy. Intensive Care Med. (2020) 46:383–400. doi: 10.1007/s00134-019-05872-y, 31965266 PMC7067741

[17] Singer M Deutschman CS Seymour CW Shankar-Hari M Annane D Bauer M . The third international consensus definitions for sepsis and septic shock (sepsis-3). JAMA. (2016) 315:801–10. doi: 10.1001/jama.2016.0287, 26903338 PMC4968574

[18] Kellum JA Lameire N Aspelin P Barsoum RS Burdmann EA Goldstein SL . Kidney disease: improving global outcomes (KDIGO) acute kidney injury work group. KDIGO clinical practice guideline for acute kidney injury. Kidney Int Suppl. (2012) 2:1–138. doi: 10.1038/kisup.2012.1

[19] Deo RC. Machine learning in medicine. Circulation. (2015) 132:1920–30. doi: 10.1161/CIRCULATIONAHA.115.001593, 26572668 PMC5831252

[20] Handelman GS Kok HK Chandra RV Razavi AH Lee MJ Asadi H. Edoctor: machine learning and the future of medicine. J Intern Med. (2018) 284:603–19. doi: 10.1111/joim.12822, 30102808

[21] Chang SI Ghafariasl P Aryal B. A review of artificial intelligence impacting statistical process monitoring and future directions. Comput Ind Eng. (2026) 216:111989. doi: 10.1016/j.cie.2026.111989

[22] Mohammadamin Ansari Khoushabar PG. Advanced meta-ensemble machine learning models for early and accurate sepsis prediction to improve patient outcomes. arXiv. (2024) 2024:8107. doi: 10.48550/arXiv.2407.08107

[23] Zuk A Bonventre JV. Acute kidney injury. Annu Rev Med. (2016) 67:293–307. doi: 10.1146/annurev-med-050214-013407, 26768243 PMC4845743

[24] Kaddourah A Basu RK Bagshaw SM Goldstein SL. Epidemiology of acute kidney injury in critically ill children and young adults. N Engl J Med. (2017) 376:11–20. doi: 10.1056/NEJMoa1611391, 27959707 PMC5322803

[25] Chiofolo C Chbat N Ghosh E Eshelman L Kashani K. Automated continuous acute kidney injury prediction and surveillance: a random forest model. Mayo Clin Proc. (2019) 94:783–92. doi: 10.1016/j.mayocp.2019.02.009, 31054606

[26] Bellomo R Kellum JA Ronco C Wald R Martensson J Maiden M . Acute kidney injury in sepsis. Intensive Care Med. (2017) 43:816–28. doi: 10.1007/s00134-017-4755-7, 28364303

[27] Bouch DC Thompson JP. Severity scoring systems in the critically ill. Contin Educ Anaesth Crit Care Pain. (2008) 8:181–5. doi: 10.1093/bjaceaccp/mkn033

[28] Patel P Gupta S Patel H Bashar MA. Assessment of APACHE II score to predict ICU outcomes of patients with AKI: a single-center experience from Haryana, North India. Indian J Crit Care Med. (2022) 26:276–81. doi: 10.5005/jp-journals-10071-24142, 35519933 PMC9015919

[29] Achanti A Szerlip HM. Acid-base disorders in the critically ill patient. Clin J Am Soc Nephrol. (2023) 18:102–12. doi: 10.2215/CJN.04500422, 35998977 PMC10101555

[30] Liao J Xiao X Lu D Wang M Huang W. The role of albumin-corrected anion gap as a predictor of all-cause mortality in patients with sepsis-AKI: a propensity score-matched cohort study. PLoS One. (2025) 20:e0327914. doi: 10.1371/journal.pone.0327914, 40680007 PMC12274010

[31] Zhu A Whitlock RH Ferguson TW Nour-Mohammadi M Komenda P Rigatto C . Metabolic acidosis is associated with acute kidney injury in patients with CKD. Kidney Int Rep. (2022) 7:2219–29. doi: 10.1016/j.ekir.2022.07.005, 36217527 PMC9546743

[32] Wu H Liao B Cao T Ji T Huang J Ma K. Diagnostic value of RDW for the prediction of mortality in adult sepsis patients: a systematic review and meta-analysis. Front Immunol. (2022) 13:997853. doi: 10.3389/fimmu.2022.997853, 36325342 PMC9618606

[33] Gu Q Huang P Yang Q Meng X Zhao M. A nomogram to predict 28-day mortality in patients with sepsis combined coronary artery disease: retrospective study based on the MIMIC-III database. Front Med (Lausanne). (2024) 11:1433809. doi: 10.3389/fmed.2024.1433809, 39296895 PMC11408215

[34] Zhou H. Total bilirubin level is associated with acute kidney injury in neonates admitted to the neonatal intensive care units: based on MIMIC-III database. Eur J Pediatr. (2024) 183:4235–41. doi: 10.1007/s00431-024-05682-5, 38990386 PMC11413182

[35] Mohammadi Kebar S Hosseini Nia S Maleki N Sharghi A Sheshgelani A. The incidence rate, risk factors and clinical outcome of acute kidney injury in critical patients. Iran J Public Health. (2018) 47:1717–24. 30581789 PMC6294852

[36] Wu Y Wu C Cheng C Tsai S. Severe hyperbilirubinemia is associated with higher risk of contrast-related acute kidney injury following contrast-enhanced computed tomography. PLoS One. (2020) 15:e0231264. doi: 10.1371/journal.pone.0231264, 32294106 PMC7159198

[37] Lee SJ Shin SW. Mechanisms, pathophysiology, and management of obesity. N Engl J Med. (2017) 376:1491–2. doi: 10.1056/NEJMc1701944 28406283

[38] Pepper DJ Sun J Welsh J Cui X Suffredini AF Eichacker PQ. Increased body mass index and adjusted mortality in ICU patients with sepsis or septic shock: a systematic review and meta-analysis. Crit Care. (2016) 20:181. doi: 10.1186/s13054-016-1360-z, 27306751 PMC4908772

[39] Schetz M De Jong A Deane AM Druml W Hemelaar P Pelosi P . Obesity in the critically ill: a narrative review. Intensive Care Med. (2019) 45:757–69. doi: 10.1007/s00134-019-05594-1, 30888440

[40] Li Q Lv H Chen Y Shen J Shi J Zhou C. Development and validation of a machine learning predictive model for cardiac surgery-associated acute kidney injury. J Clin Med. (2023) 12:10.3390/jcm12031166. doi: 10.3390/jcm12031166, 36769813 PMC9917969

[41] Hu H An S Sha T Wu F Jin Y Li L . Association between dexmedetomidine administration and outcomes in critically ill patients with sepsis-associated acute kidney injury. J Clin Anesth. (2022) 83:110960. doi: 10.1016/j.jclinane.2022.110960, 36272399

[42] Loomba RS Villarreal EG Dhargalkar J Rausa J Dorsey V Farias JS . The effect of dexmedetomidine on renal function after surgery: a systematic review and meta-analysis. J Clin Pharm Ther. (2022) 47:287–97. doi: 10.1111/jcpt.13527, 34510502

[43] Gao X Wu Y. Perioperative acute kidney injury: the renoprotective effect and mechanism of dexmedetomidine. Biochem Biophys Res Commun. (2024) 695:149402. doi: 10.1016/j.bbrc.2023.149402, 38159412

[44] Zhang Z Ho KM Hong Y. Machine learning for the prediction of volume responsiveness in patients with oliguric acute kidney injury in critical care. Crit Care. (2019) 23:112. doi: 10.1186/s13054-019-2411-z, 30961662 PMC6454725

[45] Zimmerman LP Reyfman PA Smith ADR Zeng Z Kho A Sanchez-Pinto LN . Early prediction of acute kidney injury following ICU admission using a multivariate panel of physiological measurements. BMC Med Inform Decis Mak. (2019) 19:16. doi: 10.1186/s12911-019-0733-z, 30700291 PMC6354330

[46] Liu J Wu J Liu S Li M Hu K Li K. Predicting mortality of patients with acute kidney injury in the ICU using XGBoost model. PLoS One. (2021) 16:e0246306. doi: 10.1371/journal.pone.0246306, 33539390 PMC7861386

[47] Song X Liu X Liu F Wang C. Comparison of machine learning and logistic regression models in predicting acute kidney injury: a systematic review and meta-analysis. Int J Med Inform. (2021) 151:104484. doi: 10.1016/j.ijmedinf.2021.104484, 33991886

