# Supplementary material for: Machine learning model for predicting the risk of AKI in early hemodynamically stable sepsis patients: a study based on the MIMIC IV database
Source: Front Med (Lausanne). 2026 May 26;13:1846554. doi: 10.3389/fmed.2026.1846554 (PMC13246684; doi:10.3389/fmed.2026.1846554)
Supplement: Supplementary file 1 [file Data_Sheet_1.docx]

**Supplemental informations**

| **Table S1. Performance comparison of the machine learning models** | | | | | | | | | | | | | | | | |
| --- | --- | --- | --- | --- | --- | --- | --- | --- | --- | --- | --- | --- | --- | --- | --- | --- |
|  | |  | | AUC | | SEN | | SPE | | Kappa | ACC | F1 | MCC | | Brier | |
| Imputed dataset | LR | | 0.785 | | 0.878 | | 0.512 | | 0.414 | | 0.743 | 0.811 | | 0.426 | | 0.178 |
|  | RF | | 0.842 | | 0.852 | | 0.684 | | 0.544 | | 0.790 | 0.837 | | 0.544 | | 0.151 |
|  | XGBoost | | 0.845 | | 0.865 | | 0.682 | | 0.556 | | 0.797 | 0.843 | | 0.558 | | 0.149 |
|  | GBM | | 0.844 | | 0.856 | | 0.694 | | 0.556 | | 0.796 | 0.841 | | 0.557 | | 0.150 |
|  | CART | | 0.786 | | 0.877 | | 0.651 | | 0.544 | | 0.794 | 0.843 | | 0.548 | | 0.161 |
| Non-imputed dataset | LR | | 0.790 | | 0.891 | | 0.513 | | 0.428 | | 0.742 | 0.807 | | 0.445 | | 0.178 |
|  | RF | | 0.841 | | 0.861 | | 0.677 | | 0.549 | | 0.789 | 0.831 | | 0.551 | | 0.154 |
|  | XGBoost | | 0.843 | | 0.865 | | 0.666 | | 0.543 | | 0.787 | 0.831 | | 0.546 | | 0.151 |
|  | GBM | | 0.843 | | 0.856 | | 0675 | | 0.541 | | 0.785 | 0.828 | | 0.543 | | 0.151 |
|  | CART | | 0.811 | | 0.856 | | 0.670 | | 0.536 | | 0.783 | 0.827 | | 0.539 | | 0.164 |

AUC: area under the curve; SEN: Sensitivity; SPE: Specificity; ACC: accuracy; MCC: Matthews correlation coefficient.


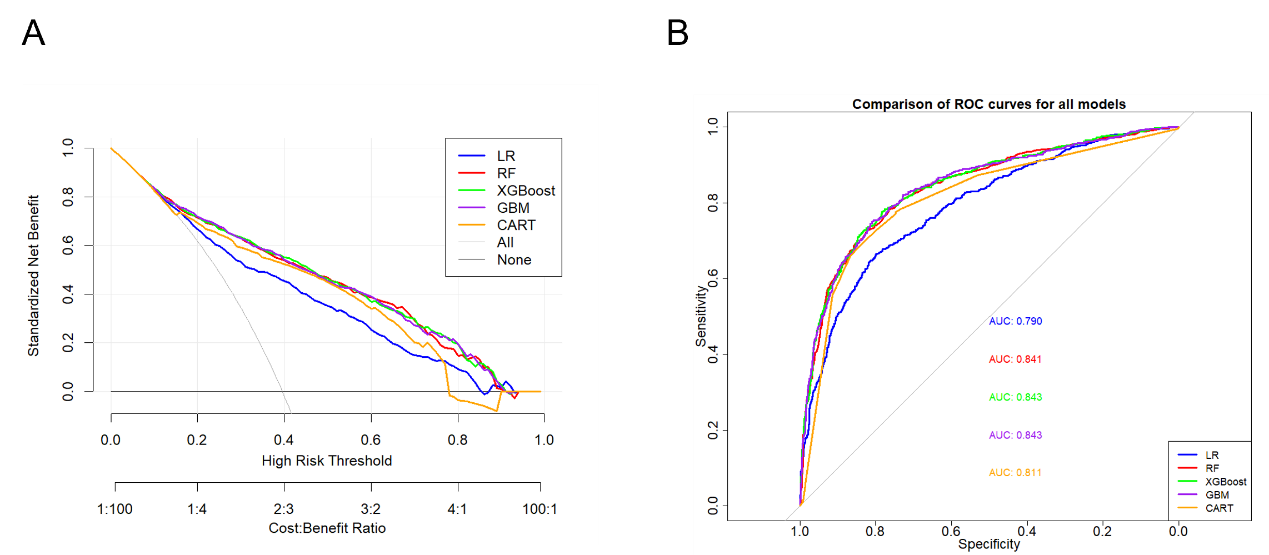


**Figure S1. ROC curves and DCA curves of the ML models in non-imputed dataset**

**(A)** DCA curves of the of the ML models in non-imputed dataset. The X-axis represents the threshold probability of clinical decisions, and the Y-axis represents the net benefit. The first horizontal axis represents the threshold probability value, and the second horizontal axis represents the loss-benefit ratio. A horizontal line indicates that all samples do not intervene, while a diagonal line indicates that all samples intervene.  **(B)** ROC curve of the of the ML models in non-imputed dataset. The X-axis represents the false positive rate (1 - specificity), and the Y-axis represents the true positive rate (sensitivity). AUC: area under the curve.
